# Commissioning and quality assurance of Dynamic WaveArc irradiation

**DOI:** 10.1120/jacmp.v16i2.5080

**Published:** 2015-03-08

**Authors:** Sayaka Sato, Yuki Miyabe, Kunio Takahashi, Masahiro Yamada, Mitsuhiro Nakamura, Yoshitomo Ishihara, Kenji Yokota, Shuji Kaneko, Takashi Mizowaki, Hajime Monzen, Masahiro Hiraoka

**Affiliations:** ^1^ Department of Radiation Oncology and Image‐applied Therapy Graduate School of Medicine, Kyoto University Kyoto; ^2^ Business Development Department Medical System Engineering Section, Mitsubishi Heavy Industries, Ltd. Hiroshima; ^3^ Department of Radiation Oncology Graduate school of Medical Science, Kinki University Osaka Japan

**Keywords:** Vero4DRT, Dynamic WaveArc, commissioning

## Abstract

A novel three‐dimensional unicursal irradiation technique “Dynamic WaveArc” (DWA), which employs simultaneous and continuous gantry and O‐ring rotation during dose delivery, has been implemented in Vero4DRT. The purposes of this study were to develop a commissioning and quality assurance procedure for DWA irradiation, and to assess the accuracy of the mechanical motion and dosimetric control of Vero4DRT. To determine the mechanical accuracy and the dose accuracy with DWA irradiation, 21 verification test patterns with various gantry and ring rotational directions and speeds were generated. These patterns were irradiated while recording the irradiation log data. The differences in gantry position, ring position, and accumulated MU ($E_{G},E_{R}$, and $E_{\text{MU}}$, respectively) between the planned and actual values in the log at each time point were evaluated. Furthermore, the doses delivered were measured using an ionization chamber and spherical phantom. The constancy of radiation output during DWA irradiation was examined by comparison with static beam irradiation. The mean absolute error (MAE) of $E_{G}$ and $E_{R}$ were within 0.1° and the maximum error was within 0.2°. The MAE of $E_{\text{MU}}$ was within 0.7 MU, and maximum error was 2.7 MU. Errors of accumulated MU were observed only around control points, changing gantry, and ring velocity. The gantry rotational range, in which $E_{\text{MU}}$ was greater than or equal to 2.0 MU, was not greater than 3.2%. It was confirmed that the extent of the large differences in accumulated MU was negligibly small during the entire irradiation range. The variation of relative output value for DWA irradiation was within 0.2%, and this was equivalent to conventional arc irradiation with a rotating gantry. In conclusion, a verification procedure for DWA irradiation was designed and implemented. The results demonstrated that Vero4DRT has adequate mechanical accuracy and beam output constancy during gantry and ring rotation.

PACS number: 87

## I. INTRODUCTION

The Vero4DRT (MHI‐TM2000, Mitsubishi Heavy Industries, Ltd., Hiroshima, Japan, and BrainLAB, Feldkirchen, Germany) is a unique image‐guided radiotherapy system,^1^, ^2^, ^3^ consisting of an O‐ring gantry that is designed to rotate $\pm 185^{\circ }$ around a patient (gantry rotation) and $\pm 60^{\circ }$ around its vertical axis (ring rotation). The Vero4DRT has two imaging units with $\pm 45^{\circ }$ against MV beam axis, each consisting of a kV X‐ray tube and a flat panel detector. It is possible to obtain kV images in two orthogonal directions simultaneously. The O‐ring gantry has a high rigid design by this O‐ring shape, and this system has the high accuracy of a static MV beam positioning.^1^, ^3^

In Vero4DRT, the O‐ring gantry rotates around the vertical axis instead of rotating around the treatment couch. Noncoplanar irradiation can be performed without moving a patient by ring rotation. Furthermore, irradiation during continuous ring rotation can be performed mechanistically. Using the mechanical features of the O‐ring gantry, a novel three‐dimensional unicursal irradiation method, called “Dynamic WaveArc” (DWA), which uses simultaneous and continuous gantry and ring rotation during dose delivery, has been developed.^(4)^ DWA is an advanced conformal arc irradiation technique allowing more flexibility in irradiation direction, and it enables efficient dose delivery avoiding the organ at risk (OAR). In the initial investigation with a planning study,^(4)^ a comparison of the treatment plan in pancreatic cancer by DWA, a conventional dynamic conformal arc irradiation, and intensity‐modulated radiation therapy (IMRT) was performed. It was found that the dose‐volume data of DWA were comparable to those of IMRT in relation to both the target and the OARs, and the monitor unit (MU) was 22.1% lower than IMRT. In some clinical cases, large advantages with improvement in dose distribution and shortening of treatment time can be expected.

The mechanical control required for DWA irradiation has been implemented in Vero4DRT and experimental validation with an actual machine became possible. In this study, we developed a commissioning and quality assurance (QA) procedure for DWA irradiation, and verified the accuracy of the mechanical motion and dosimetric control of Vero4DRT.

## II. MATERIALS AND METHODS

### A. Implementation of DWA

DWA is a rotational irradiation method that employs simultaneous and continuous gantry and ring rotation during dose delivery. Specifically, the ring rotation changes in the negative or positive direction continuously while the gantry rotates in one direction (maximum gantry rotation range is 360°). Thus, this irradiation is carried out with the trajectory of the wave to the patient's body. Compared with typical rotation irradiation methods, such as dynamic conformal arc and volumetric‐modulated arc therapy (VMAT) which use only gantry rotation, DWA irradiation has more flexibility in terms of the beam incidence direction, and enables the concentrating of the dose to a target while avoiding the OARs. In DWA irradiation, the output dose per gantry and ring rotational angle are designed to be constant, and the dose rate is adjusted in accordance with gantry and ring operation. A synchronized trajectory control between gantry rotation and ring rotation is implemented in Vero4DRT, and an irradiation beam axis can be passed along with the specified tracks in DWA irradiation. In this trajectory control, the ratio of ring rotation per unit time to gantry rotation per unit time in each segment is maintained constant. In the case of changing the speed of gantry or ring, the gantry and ring rotation is decelerated and stopped at a moment just before the control point, and then accelerated to the specified speed. At the same time, to suppress dose errors, the dose rate is reduced at the moment of stopping machine motion and increased again between segments.

In terms of the mechanical restriction on a specification of Vero4DRT with DWA irradiation, maximum and minimum of gantry rotational speed, ring rotational speed, and dose rate are $6.0^{\circ }/s$ and $0.1^{\circ }/s,2.5^{\circ }/s$ and $0.1^{\circ }/s$, and 400 MU/min and 150 MU/min, respectively.

### B. Commissioning of DWA

Based on the characteristics of the DWA irradiation method described in Material & Methods section A, verification tests of the mechanical accuracy and the dose accuracy were performed. These tests were designed to evaluate accuracy related to ring rotation, the synchronous control of gantry and ring rotation, variations in gantry and ring rotation speed between segments, and the control of the dose rate variations in a short time.

#### B.1 Verification experiment and irradiation patterns

**Figure 1:**
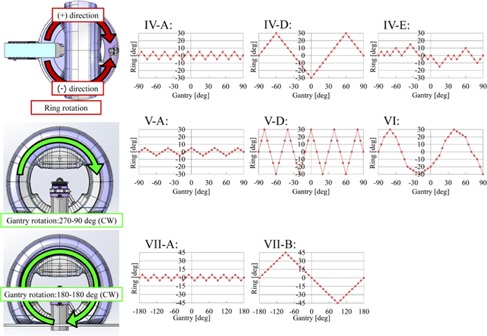
Diagrams of gantry and ring rotational trajectories in several verification patterns. The horizontal axis and vertical axis in this graph represent the gantry angle and ring angle, respectively. Groups IV, V, and VI were performed with reciprocating motion of ring rotation in the positive or negative direction simultaneously with the gantry rotating from 270° to 90° (CW direction). The patterns of Group IV feature the same ring rotational speed and different numbers of stopping points in mechanical motion, to assess the effects of the number of stopping points. The patterns of Group V feature the same number of stopping points in mechanical motion and different ring rotational speeds, to assess the effects of ring rotational speed. The patterns of Group VI feature the gantry and ring rotational speed changing during DWA irradiation, to assess the effects of gantry and ring rotational speed. The patterns of Group VII feature ring rotations of $\pm 5^{\circ }$ and $\pm 45^{\circ }$ during 360° gantry rotation, to determine the effects of the combination of fast gantry rotation and ring rotation.

The list of verification patterns is shown in Table 1, and diagrams of some of the patterns are shown in Fig. 1. The original DWA irradiation verification patterns were created as follows. Group I was ‘conventional’ arc irradiation, rotating the gantry. Groups II and III were patterns with a stationary gantry angle and a rotating ring, with a rotational range of 40°; the delivery accuracy during ring rotation was then verified. Groups IV to VII concerned the patterns of DWA irradiation with various gantry and ring rotational directions and speeds. In each group, all patterns had the same setting MU and dose rate. These verification test irradiations could be performed by reading the csv files describing the original verification patterns input into the Vero4DRT

**Figure 2:**
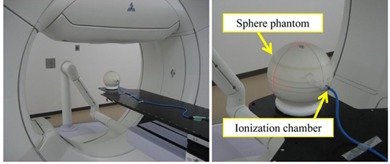
Experimental setup for output measurements.

The output values for the verification patterns of Table 1 were measured with a Farmer‐type ionization chamber (PTW30013, PTW, Freiburg, Germany) set at the center of a water‐equivalent sphere phantom with a diameter of 20 cm (Taisei Medical Co., Ltd., Osaka, Japan; Fig. 2). The composition of the material of this phantom was H (11.6%) and C (88.4%), and the density was $1.000\;g/{\text{cm}}^{3}$. It is possible to measure the output dose with less directional dependence for the three‐dimensionally high flexible DWA irradiation beam.

**Table 1:** Verification patterns of DWA irradiation

*Group*		*Irradiation Pattern*	*Gantry Angle (°)*	*Ring Angle (°)*	*Gantry Speed (°/s)*	*Ring Speed (°/s)*	*Dose Rate (MU/min)*	*Total MU (MU)*	*Point of Motion Interrupt*	*Output Measurement*
I		• gantry rotation • static ring angle	270→90	±0	2.5	0.0	333	400	0	°
II	A B C D	• static gantry angle • ring rotation	45	−30→+10 −10→+30 +30→−10 +10→−30	0.0	2.5	375	100	0	°
III	A B C D	• static gantry angle • ring rotation	90	−30→+10 −10→+30 +30→−10 +10→−30	0.0	2.5	375	100	0	°
IV	A B C D E	• DWA irradiation • ring rotation direction was changed • the number of motion interrupt point was changed	270→90	±5 ±10 ±15 ±30 ±5−15	2.5	2.5	333	400	18 9 6 3 18	°
V	A B C D	• DWA irradiation • ring speed and direction were changed	270→90	±5 ±10 ±15 ±30	0.8	0.4 0.8 1.2 2.4	160	600	9	°
VI		• DWA irradiation • gantry speed, ring speed and direction were changed	270→90	±30	1.2−3.0	1.0−2.5	160−400	400	12	°
VII	A B	• DWA irradiation • ring rotation direction was changed	180→180 (CW)	±5 ±45	5.0	2.5	333	400	18 2	Log analysis only

#### B.2 Evaluation of DWA delivery accuracy by irradiation log analysis

The planned and actual values of the gantry position, ring position, and accumulated MU each time were recorded as an irradiation log in the Vero4DRT during each irradiation test listed in Table 1. The planned values were values of the gantry position, ring position, and accumulated MU input into the Vero4DRT They were extracted from the csv files describing the original verification patterns. The actual value was a record of the actual motion of the Vero4DRT The actual values of gantry angle and ring angle were recorded using a rotary encoder. The actual values of the accumulated MU were recorded values that amplified the current of the internal chamber, and these analog data were converted to digital data. These data, planned and actual values of the gantry position, ring position, and accumulated MU, were recorded every 20 ms using the same controller. Though these log data in this study were output in text format using the service function of the manufacturer, it can be also obtained via the general function of the Vero4DRT. The differences between the planned and actual values of gantry position ($E_{G}$), ring position ($E_{R}$), and accumulated MU ($E_{\text{MU}}$) at every 20 ms were calculated. Furthermore, the positive maximum value, negative maximum value, and mean absolute error (MAE) of $E_{G},E_{R}$, and $E_{\text{MU}}$ were evaluated.

**Figure 3:**
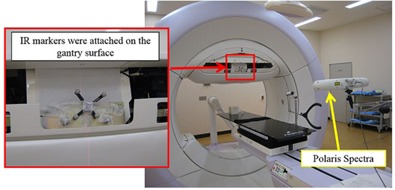
Experimental setup with Polaris Spectra.

**Figure 4:**
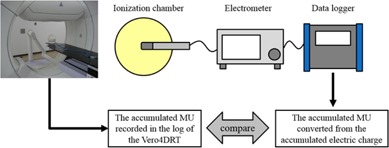
Schema of the independent measurement system of the accumulated MU.

To verify the details of the data recorded in the DWA irradiation log, the mechanical motion of the gantry and ring and accumulated MU were measured using external measuring devices that operate independently of the Vero4DRT system. Gantry and ring position were verified by Polaris Spectra (Northern Digital Inc., Waterloo, ON). The Polaris Spectra was placed in front of the Vero4DRT. Infrared (IR) markers were attached to the surface of the gantry head, and the motion trajectories of the IR markers were measured by the Polaris Spectra (Fig. 3). Gantry and ring trajectories were determined from the trajectory of the IR markers' positions. The sampling interval for data with the Polaris Spectra was ∼33 ms. The Polaris Spectra system volumetric accuracy was 0.25 mm RMS within this measurement.^(5)^ The accuracy of the accumulated MU recorded in the log was verified with a Farmer‐type ionization chamber (PTW30013), an electrometer (RAMTEC Smart, Toyo Medic Co., Ltd., Tokyo, Japan), and a data logger (MEMORY HiCORDER MR8880, HIOKI E.E. Co., Tokyo, Japan). The data logger, a commercially available product, could record the sequential output values of electric charge from the ionization chamber. A comparison was performed between the accumulated MU (in MU) converted from the accumulated electric charge (in nC) measured by the ionization chamber, the electrometer, and the data logger and the accumulated MU (in MU) recorded in the log (Fig. 4). The sampling intervals of the data with the measurement system using the data logger and the log data of the Vero4DRT were 100 ms and 20 ms, respectively.

#### B.3 X‐ray output constancy during DWA irradiation

The delivered doses for the verification patterns of Table 1 were measured placing the Farmer‐type ionization chamber at the center of the sphere phantom (Fig. 2), and the constancy of radiation output during DWA was examined. The ionization chamber was placed horizontally against the couch in the direction that the tip of the chamber toward the gantry side. In these measurements, the field size was set at $5\times 5\;{\text{cm}}^{2}$. The variation in machine output was assessed by comparison of relative output values during the test irradiation including DWA irradiation patterns (verification Patterns I to VI), with a static beam (gantry and ring angle at 0°) irradiation with the same MU of the DWA irradiation. The irradiation beam of verification Pattern VII passed through the carbon fiber couch top, so these measurements might have contained uncertainties in dose reduction due to the couch. The water‐equivalent sphere phantom used in this study was designed to be placed on the couch. Consequently, it could not be placed so as not to pass through the couch with respect to the beam from a posterior angle. To accurately confirm the DWA irradiation dose accuracy in this study, it was considered necessary to assess dose accuracy under conditions that removed as much uncertainty as possible in test patterns with full gantry rotation (360°). Thus, output measurements were not performed in the Pattern VII.

## III. RESULTS

### A. Accuracy of DWA delivery

**Figure 5:**
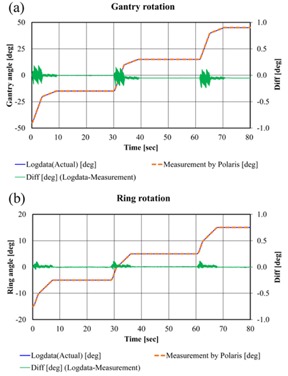
Comparison between actual log data and data measured with the Polaris Spectra. (a) Gantry rotation paused every 30° during gantry rotation from 315° to 45° with a fixed ring angle (of 0°). (b) Ring rotation paused every 10° during ring rotation from 345° to 15° with a fixed gantry angle (of 0°).

The accuracy of the gantry and ring positions recorded in the irradiation log was verified by comparing values measured using the Polaris Spectra. Figure 5 shows an example of the results. The differences in gantry rotation and ring rotation between values measured by the Polaris Spectra and the actual value recorded in the log were consistent, within $\pm 0.3^{\circ }$ and $\pm 0.2^{\circ }$, respectively. It was confirmed in advance that trajectory information on the ring and gantry that had been recorded in the log was appropriate.

**Figure 6:**
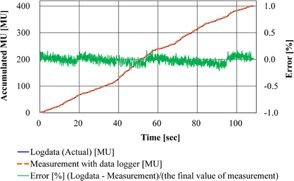
Comparison of the actual log data of accumulated MU and the amount of measured electric charge for verification Pattern VI. The percentage of error value (%) was determined relative to the last value of the accumulated measured electric charge.

Figure 6 shows a comparison of the measurement values by the data logger and the actual values recorded in the log. These errors were within ±0.21% of the total MU. These results demonstrated that the machine rotational motion and irradiated dose were recorded in the log with subdegree and subpercentage accuracy.

Table 2 shows the results of the log analysis. $E_{G}$ and $E_{R}$ were within $\pm 0.2^{\circ }$ and $\pm 0.1^{\circ }$, and the maximum values of the mean absolute error (MAE) of each error were 0.1° and 0.1°, respectively. The actual values of gantry and ring mechanical motion were delayed briefly compared with a planned value. These delays were less than 60 ms and considered to be mechanical response delays.

**Figure 7:**
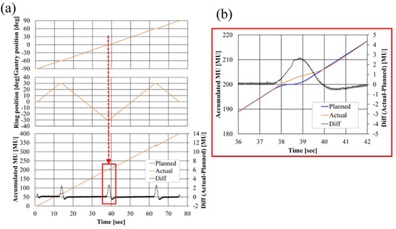
Log data of Pattern IV‐D. (a) Log of ring angle, gantry angle, and accumulated MU. Because $E_{R}$ and $E_{G}$ in Pattern IV‐D were negligible, only the results of the actual gantry and ring angle values are displayed. For the accumulated MU, both the planned and actual values are displayed. (b) Enlarged view of accumulated MU at the point at which ring rotation was reversed (square region of (a)). This point had the maximum $E_{\text{MU}}$ for this pattern.

E_MU_ were within ±2.7 MU, and the maximum value of MAE was 0.7 MU. The $E_{\text{MU}}$ of patterns with DWA irradiation (Patterns IV, VI, VII) had positive maximum errors over 2.0 MU, and MAEs were from 0.2 MU to 0.7 MU for these verification patterns. Patterns I, II, and III had constant rotational speed and no reversal of ring rotation, but Patterns IV, VI, and VII have stopping points for mechanical motion when the ring rotational direction was changed and rotational speeds were high. Regardless of gantry and ring positions, differences in accumulated MU were noticeable around these points. As an example, the detailed log data of Pattern IV‐D that had maximum $E_{\text{MU}}$ (2.7 MU) are shown in Fig. 7. This result demonstrates that the dose rate was adjusted according to the gantry and ring rotational speed, to decrease dose errors. Pattern VI also had the maximum $E_{\text{MU}}$ (2.7 MU) at the point of changing ring rotational speed and stopping mechanical motion. In the region not including the area around the point of stopping the mechanical motion, the actual value of accumulated MU was in good agreement with the planned value. The results of Pattern V showed that positive $E_{\text{MU}}$ increased gradually as ring rotational speeds increased, with a linear correlation.

**Figure 8:**
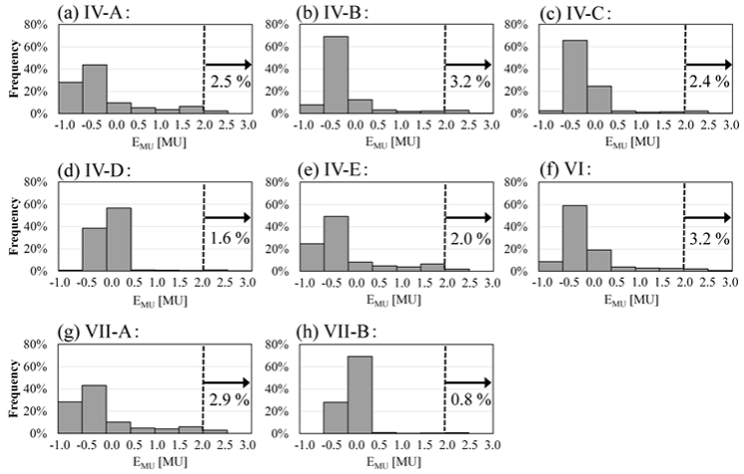
Frequency distribution of $E_{\text{MU}}$ in Patterns IV, VI and VII. The percentage frequencies for which $E_{\text{MU}}$ was greater than or equal to 2.0 MU are indicated in each graph.

The frequency distributions of $E_{\text{MU}}$ in eight verification patterns in which the maximum value was greater than 2.0 MU (Patterns IV, VI, VII) are shown in Fig. 8. In the irradiation of each pattern, the percentage of gantry rotational range of absolute $E_{\text{MU}}$ less than 1.0 MU was 86.6% or higher, and the percentages for which the differences were greater than or equal to 2.0 MU were from 0.8% to 3.2%.

**Table 2:** Negative and positive maximum values and mean absolute errors (MAE) of $E_{G},E_{R}$, and $E_{\text{MU}}$ for each verification pattern as evaluated by irradiation log analysis

*Group*		$E_{G}$ (°)			$E_{R}$ (°)			$E_{MU}$ *(MU)*		
		*neg*	*pos*	*MAE*	*neg*	*pos*	*MAE*	*neg*	*pos*	*MAE*
I		−0.1	0.0	0.1	0.0	0.0	0.0	−0.3	0.4	0.1
II	A	0.0	0.0	0.0	−0.1	0.0	0.1	−0.2	0.7	0.1
	B	0.0	0.0	0.0	−0.1	0.0	0.1	−0.2	0.8	0.1
	C	0.0	0.0	0.0	0.0	0.1	0.1	−0.2	0.8	0.2
	D	0.0	0.0	0.0	0.0	0.1	0.1	−0.2	0.8	0.1
III	A	0.0	0.0	0.0	−0.1	0.0	0.1	−0.2	0.7	0.1
	B	0.0	0.0	0.0	−0.1	0.0	0.1	−0.2	0.8	0.1
	C	0.0	0.0	0.0	0.0	0.1	0.1	−0.2	0.7	0.2
	D	0.0	0.0	0.0	0.0	0.1	0.1	−0.2	0.8	0.1
IV	A	−0.1	0.0	0.1	−0.1	0.1	0.1	−1.0	2.4	0.7
	B	−0.1	0.0	0.1	−0.1	0.1	0.1	−0.8	2.6	0.4
	C	−0.1	0.0	0.1	−0.1	0.1	0.1	−0.7	2.6	0.3
	D	−0.1	0.0	0.1	−0.1	0.1	0.1	−0.6	2.7	0.2
	E	−0.1	0.0	0.1	−0.1	0.1	0.1	−1.0	2.3	0.7
V	A	−0.1	0.0	0.0	0.0	0.0	0.0	−0.4	0.5	0.1
	B	−0.1	0.0	0.0	0.0	0.0	0.0	−0.1	0.7	0.1
	C	−0.1	0.0	0.0	0.0	0.0	0.0	−0.2	0.9	0.1
	D	−0.1	0.0	0.0	−0.1	0.1	0.1	−0.3	1.5	0.1
VI		−0.1	0.0	0.1	−0.1	0.1	0.1	−0.9	2.7	0.5
VII	A	−0.2	0.0	0.1	−0.1	0.1	0.1	−0.9	2.4	0.7
	B	−0.2	0.0	0.1	−0.1	0.1	0.1	−0.5	2.4	0.2

neg=negative maximum error;pos=positive maximum error;MAE=mean absolute error.

### B. X‐ray output constancy during DWA irradiation

**Figure 9:**
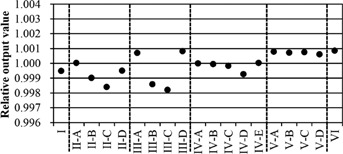
Relative output values of the verification patterns in Table 1.

Figure 9 shows the relative output values for the verification patterns in Table 1. The relative output value for conventional arc irradiation, by rotating the gantry (Pattern I), was 0.9995. The variations of relative output values during irradiation with ring rotations (Patterns II and III), and DWA irradiations (Patterns IV to VII) were within ±0.2%. There was a tendency for decreased relative output values in the cases of patterns in which the radiation beam came from the caudal direction (Patterns II‐B, C, III‐B, C).

## IV. DISCUSSION

In a previous study of the Vero4DRT, we reported that the displacement of the MV beam isocenter with various gantry and ring angles was less than 0.36 mm in the vertical, longitudinal, and lateral directions.^(3)^ This result is substantially equivalent to the isocenter positional accuracy with gantry and couch rotation in a high‐precision linac system.^6^, ^7^ Depuydt et al.^(8)^ performed star shot film analysis and compared the mechanical stability of the Vero4DRT with an L‐shaped linac. The Vero4DRT and Varian TrueBeam system showed comparable mechanical performance, in that the gantry isocenter radii were 0.12 mm and 0.09 mm, respectively, and the isocenter radii for ring/couch rotation axes were less than 0.10 mm in both systems. Sufficient mechanical accuracy in fixed‐field irradiation has been demonstrated in previous studies.

In this study, a comparison between measurement values by a device independent of the Vero4DRT and the actual value recorded in the log of Vero4DRT was also performed. For gantry angle and ring angle, the Polaris Spectra was used as an independent measurement device. It was impossible to measure the area including all of the DWA test patterns' motion (gantry and ring rotation) because the measurable volume of Polaris Spectra was limited; thus, the verification test of the accuracy of the record in the log was carried out on the simple test patterns (Fig. 5) within the range that could be measured with the Polaris Spectra. The accuracies of the gantry and ring rotation of all DWA irradiation test patterns were verified using the planned and actual values in the log of the Vero4DRT. For the accumulated MU, the ionization chamber, the electrometer, and the data logger were used as an independent measurement system (Fig. 4). It was possible to compare the planned value in the log and the measured value by the data logger directly. However, the data sampling interval of this measurement system was longer (100 ms) than the log data of the Vero4DRT (20 ms); therefore, the accuracy of the accumulated MU of all DWA irradiation test patterns was verified using the planned and actual value in the log of the Vero4DRT. In one verification pattern, it was confirmed that the error between the actual value recorded in the log and the measured value with the independent measurement system was sufficiently small (Fig. 6). The analysis of the irradiation log results showed that although the actual values of gantry and ring mechanical motion were delayed momentarily compared with the planned values, $E_{G}$ and $E_{R}$ were negligible with all patterns. The same verification tests using log analyses have been performed for gantry arc rotation in other linac systems. It has been reported that the gantry maximum error during VMAT was $0.34\pm 0.6^{\circ }$.^(9)^ In studies of the RapidArc, by log analysis, the gantry angle deviation was less than 1° from the planned value,^(10)^ and the maximum gantry position discrepancy was $0.9\pm 0.2^{\circ }$.^(11)^ The mechanical rotational accuracy of Vero4DRT is controlled with high precision and the value of $E_{G}$ in this study was considerably lower than the precision reported in previous studies. Moreover, $E_{R}$ was of the same order as $E_{G}$, so the ring rotation accuracy of Vero4DRT can be regarded as comparable to that of gantry rotation. Thus, ring rotation irradiation can be realized with a similarly high mechanical precision as conventional gantry rotation irradiation.

$E_{\text{MU}}$ was slightly increased in the DWA irradiation at the control points to change the speed of gantry or ring rotation. At these points, the beam is intended to be turned off at the same time as the mechanical motion is stopped, and to be turned on again when gantry or ring motion resumes. However, in the actual machine, dose rate adjustment is performed instead of the beam switching, because variations in output can be increased by switching the beam off and on again within a short time. At the point when the beam is turned off, the dose rate is, in fact, reduced to ∼100 MU/min. Thus, the differences between planned and actual values of accumulated MU occurred during this short time.

As the rotational speed of the gantry and ring increase, the mechanical braking time at the point of changing speed becomes longer and $E_{\text{MU}}$ is increased. Thus, it is preferred that the verification patterns with the fastest gantry and ring speeds are generated and the irradiation tests are performed under stricter conditions. However, $E_{\text{MU}}$ remained lower than 3.0 MU even when the gantry and ring were rotating at their maximum speeds in all verification patterns in this study. From the results in Fig. 8, it was confirmed that the extent of differences of greater than or equal to 2.0 MU was within 3.2% during the entire irradiation gantry range. These deviations in the accumulated MU have negligible effects on the actual dose.

In the results of relative output measurement, the variations of relative output values measured at the isocenter were within ±0.2% in all verification patterns in this study. These variations were within the allowable value of 2% dose variations for stereotactic radiosurgery in arc rotation mode reported by the AAPM TG‐142.^(12)^ Relative output values decreased when the ring rotational range was $-10^{\circ }$ to $+30^{\circ }$ (a pattern with a wide range of irradiation from the inferior direction: Patterns II‐B, C, III‐B, C). This was presumed to be due to the directional dependence of the ionization chamber response. When measurements of the dose verification patterns of DWA irradiations were performed, the relative output measurement of static (not arc) beam for the noncoplanar beam against the ionization chamber was also performed as a basic measurement test. The phantom and the chamber were set in the same arrangement as in the DWA irradiation test. In the results, measured relative output values in the case of the ionization chamber irradiated with $5\times 5\;{\text{cm}}^{2}$ field size, from the direction of the chamber's stem (gantry: 90° and ring: $+30^{\circ }$, gantry: 270° and ring: $-10^{\circ }$) were reduced by ~ 0.7% compared with the measured value of the ring angle (0°). It was presumed that the decreases in the relative output value of the Patterns II‐B, C, III‐B, C in Fig. 9, compared with the other test patterns, were caused by the incident angle of the beam against the ionization chamber. The relative output values varied little in these patterns, as a whole, and the effect of variation of mechanical motion speed was small. In assessing the output constancy during DWA irradiation, we used a custom‐made spherical phantom to eliminate any influence of angular dependence on the measurement. The sphere phantom is useful for commissioning and QA measurements of a noncoplanar beam, including DWA irradiation.

Furthermore, in this study, the verification of the mechanical accuracy and the dose accuracy from a range of gantry of anterior angle in the DWA irradiation test (Patterns IV, V, VI) were performed due to the limitation of the setting of a sphere phantom, and it was confirmed that the differences in mechanical motion and the variation in the relative output value were small. Additionally, in Pattern VII, we verified that in mechanical motion with full gantry rotation (360°), the differences in mechanical motion were small. From these results, it would be expected that the variation of the relative output value of the irradiation from the posterior range also would be small.

In some simulation studies, plans with couch rotation have shown greater target dose conformity and decreased the dose to OARs and normal tissue versus a normal coplanar plan.^13^, ^14^, ^15^ A benefit similar to that of a couch rotational plan can be achieved using DWA irradiation. From the results of the current study, the ring rotation of Vero4DRT has equivalent or better mechanical accuracy than the couch rotation of an L‐shaped linac. Additionally, it is not necessary to move the patient during DWA irradiation. Furthermore, during treatment with the Vero4DRT, the ring can be rotated automatically and entering the treatment room is not required. Thus, there is no extension of treatment time and no decrease in accuracy. This is a major advantage in clinical use. In the commissioning and QA procedure developed in this study, verification was performed separately for gantry rotation, ring rotation, and the combination of gantry and ring rotation. Basically, the verification procedure for ring rotation is the same as that conventionally performed for gantry rotation. Machine commissioning for DWA irradiation requires a little additional work versus that for conventional conformal arc therapy, but it is not time consuming. A treatment planning system that corresponds to DWA irradiation is under development. After that is complete, additional commissioning related to the treatment planning system will be needed before clinical use of DWA irradiation.

V. CONCLUSIONS

Commissioning and QA procedures for DWA irradiation were designed and implemented. The results demonstrated that Vero4DRT has sufficient mechanical accuracy in the control of gantry and ring rotation and beam output constancy during DWA irradiation.

## ACKNOWLEDGMENTS

This research was supported in part by the Center of Innovation Program from the Japan Science and Technology Agency, JST, and Grants‐in‐Aid for Scientific Research from the Ministry of Education, Culture, Sports, Science, and Technology of Japan (Grants No. 20229009).
